# Sensitive responders among bacterial and fungal microbiome to pyrogenic organic matter (biochar) addition differed greatly between rhizosphere and bulk soils

**DOI:** 10.1038/srep36101

**Published:** 2016-11-08

**Authors:** Zhongmin Dai, Jiajie Hu, Xingkun Xu, Lujun Zhang, Philip C. Brookes, Yan He, Jianming Xu

**Affiliations:** 1 Institute of Soil and Water Resources and Environmental Science, College of Environmental and Resource Sciences, Zhejiang University, Hangzhou 310058, China; 2 Zhejiang Provincial Key Laboratory of Agricultural Resources and Environment, Hangzhou 310058, China

## Abstract

Sensitive responses among bacterial and fungal communities to pyrogenic organic matter (PyOM) (biochar) addition in rhizosphere and bulk soils are poorly understood. We conducted a pot experiment with manure and straw PyOMs added to an acidic paddy soil, and identified the sensitive “responders” whose relative abundance was significantly increased/decreased among the whole microbial community following PyOM addition. Results showed that PyOMs significantly (*p* < 0.05) increased root growth, and simultaneously changed soil chemical parameters by decreasing soil acidity and increasing biogenic resource. PyOM-induced acidity and biogenic resource co-determined bacterial responder community structure whereas biogenic resource was the dominant parameter structuring fungal responder community. Both number and proportion of responders in rhizosphere soil was larger than in bulk soil, regardless of PyOM types and microbial domains, indicating the microbial community in rhizosphere soil was sensitive to PyOM addition than bulk soil. The significant increased root biomass and length caused by PyOM addition, associated with physiological processes, e.g. C exudates secretion, likely favored more sensitive responders in rhizosphere soil than in bulk soil. Our study identified the responders at fine taxonomic resolution in PyOM amended soils, improved the understanding of their ecological phenomena associated with PyOM addition, and examined their interactions with plant roots.

Pyrogenic organic matter (PyOM) (also called biochar) is widely distributed in agricultural lands^1^ and fire-affected natural soils^2^, which can constitute up to 80% of total soil organic carbon^3^. PyOM has been regarded as an eco-friendly material for improving soil fertility, mitigating climate change and modifying soil microbial abundance and diversity^4^. Most previous studies have shown that PyOM plays a great role in soil acidity correction by increasing soil pH and exchangeable base cations, as well as decreasing exchangeable Al^5^,^6^,^7^. It can also optimize biogenic resource supply (e.g. C, N, P, K, etc.) for plant and microbial growth through modifications to their quantity and bioavailability^8^,^9^. Recently, the effects of PyOM on soil microbiology are gaining more and more attention, as soil biogeochemical processes are not only regulated by PyOM modified changes in chemical properties, but also in microbiological ones. The potential mechanisms of how PyOM acts on microbes may include, but are not limited to: (1) PyOM particles can serve as a habitat for microbes by protecting them from water deficiency and predation^10^; (2) the minerals and labile C fractions of PyOM can be utilized as a source of energy or nutrients for microbes^11^; (3) PyOM can change some abiotic factors that may favor some competitive microbial populations over others. e.g. pH modulation^12^; (4) PyOM-stimulated plant growth may induce the changes in microbial communities by some physiological activities^13^. Although some research has reported that how PyOM addition influenced soil microbial abundance, activity and community structure^14^, they provide little in-depth information, because of the limitations of traditional molecular biological techniques, e.g. phospholipid fatty acid and denaturing gradient gel electrophoresis based methods. Therefore, how soil microbial community responds to PyOM addition, and what the direct and/or indirect effects of PyOM on soil microbes are, need a further study for evaluating the mechanism(s) underpinning PyOM addition as an eco-friendly method for soil improvement.

Recently, high-throughput sequencing has been developing rapidly and provides a more comprehensive analysis of bacterial and fungal community structure in the complex soil environment^15^,^16^. A few recent studies have been conducted to investigate the effects of PyOM on soil bacteria based on high throughput sequencing. For example, Xu *et al*.^17^ showed that PyOM increased soil microbial α-diversity and altered the abundance of some bacteria responsible for carbon and nitrogen cycling. Khodadad *et al*.^18^ found that relative abundance of bacterial phyla *Actinobacteria* and *Gemmatimonadetes* were increased by PyOM addition to forest soils. However, PyOM also likely induces changes in fungal community, which are also critical microbial members as decomposers, plant pathogens and symbionts^19^,^20^,^21^ in the environment, and to date they have been little studied so far. Therefore, the response of fungal community to PyOM addition, as well as the difference and similarity between fungal and bacterial communities following PyOM addition are worthy of investigation.

In general, there are two types of microbial pools in soils, i.e. the microbiome in bulk soil and in rhizosphere soil. Previous studies mainly focused on PyOM effects on microbial community in bulk soils^22^,^23^, and rarely considered root physiological processes induced by PyOM addition, e.g. rhizodeposition. Some available studies have reported that PyOM had a great effect on root elongation and growth. Lehmann *et al*.^4^ concluded that root biomass was mainly increased following PyOM addition, with a maximum increase of 300%. Root growth was shown to be strongly related to physiological processes, i.e. releasing carbon exudates, subsequently influencing the microbial community and C turnover in soils^24^,^25^. With PyOM addition, the nutrients (e.g. organic C and minerals) from PyOM, associated with PyOM-corrected soil environment, can probably stimulate root growth. In turn, the carbon products secreted by facilitated growing roots, which are preferential nutrients and energy for microbial metabolism, can also affect microbial community in rhizosphere soil. In this process, PyOM would simultaneously play a critical direct and indirect role due to its unique properties. Besides the difference in microbiome between rhizosphere and bulk soils, there are also differences in microbiome between two microbial groups: (1) the microbes termed as ‘non-responders’ that are more resistant to disturbance, and (2) the microbes termed as ‘responders’ that are more sensitive to disturbance. Recently, some researchers proposed a concept of ‘responders’ to report environment modified response of soil microbiome^26^,^27^. Responder community was assumed to be more representative when research is focused on specific microbial group that is strongly affected by environmental disturbance. For example, Whitman *et al*.^27^ used this concept to demonstrate how easily mineralizable carbon induced more bacterial responders than PyOM in soils. In this sense, the responders of microbial community in response to PyOM addition were speculated to be different between rhizosphere and bulk soils.

In this study, we conducted a pot experiment with two types of widely-used PyOMs, a manure-derived PyOM and a straw-derived PyOM. They were added to an acidic paddy soil planted with rice cultivar (*Oryza sativa* L., indica variety Liangyoupeijiu). We selected the microbes whose relative abundance increase/decrease significantly by more than doubling in response to PyOM addition and defined them as the ‘responders’. Our objectives were to: (1) identify the responders among bacterial and fungal microbiome that are sensitive to PyOM addition at fine level of taxonomic resolution; (2) examine how the bacterial and fungal responder communities respond to PyOM addition; (3) investigate the difference and similarity of responder communities between rhizosphere and bulk soils, and examine the potential mechanisms involved.

## Results

### PyOM characteristics

The mineral nutrients, e.g. ash, total P and total base cations, in manure PyOM were much higher than in straw PyOM (Tables 1 and S1 and Fig. S1), which was consistent with FTIR results showing that manure PyOM had more mineral functional groups (O-Si-O, P-O, C-O) than straw PyOM (Fig. S2). In addition, the peaks assigned to organic C groups, e.g. C-O-C, -COH and -COOH in straw PyOM were more apparent compared to those of manure PyOM (Fig. S2). The BET of straw PyOM (52.6 m^2^ g^−1^) was more than four times higher than that of manure PyOM (11.7 m^2^ g^−1^) (Table 1), which was consistent with SEM spectra showing there were more pore structures in straw PyOM (Fig. S1).

### PyOM effects on soil chemical parameters and plant growth

**Figure 1:**
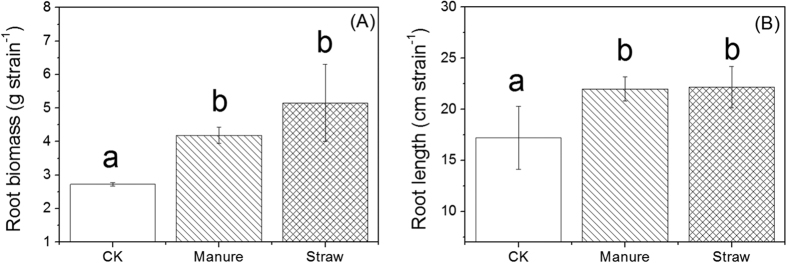
Effects of PyOMs on plant root biomass (**A**) and root length (**B**). Different letters indicate that the difference is significant at p < 0.05 probability level between different samples.

PyOM addition strongly changed soil acidity parameters and biogenic resource parameters in both rhizosphere and bulk soils, regardless of PyOM types. Regarding soil acidity parameters, PyOM addition significantly increased soil pH and BC_ex_, and decreased soil Al_ex_ (Table 2) (*p* < 0.05). For soil biogenic resource parameters, PyOM addition to some extent increased soil C_tot_, N_tot_, P_avail_, K_ex_ and DOC (Table 2). In addition, the degree of changes in acidity parameters, e.g. pH, BC_ex_ and Al_ex_ in rhizosphere soils were larger than those in bulk soils. However, the trends in some biogenic resource parameters, e.g. soil C_tot_ and P_avail_ were opposite. In addition, PyOM addition significantly (*p* < 0.05) increased plant root biomass (Fig. 1A) and root length (Fig. 1B), regardless of PyOM types.

### Bacterial and fungal taxa

**Figure 2:**
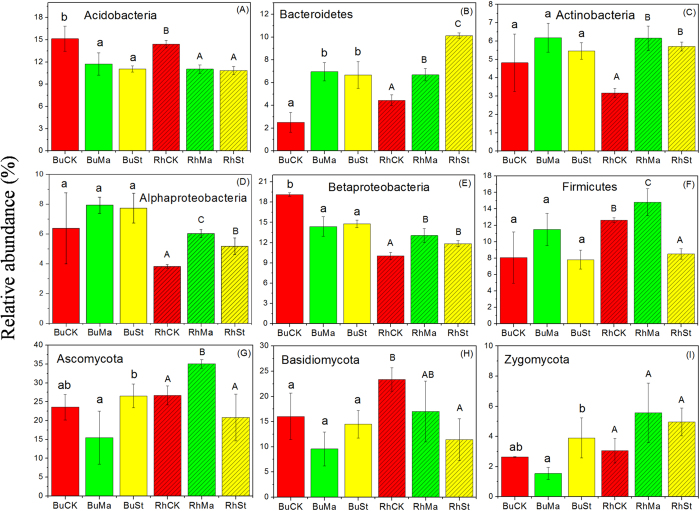
Relative abundances of the dominant bacterial and fungal taxa in PyOMs amended soils. Different letters in lower and upper case represents significant differences at p < 0.05 probability level in bulk and rhizosphere samples.

In this study, we mainly focused on the relative abundances of six bacterial taxa (i.e. *Acidobacteria, Bacteroidetes, Firmicutes, Actinobacteria, Alphaproteobacteria* and *Betaproteobacteria*) and three fungal phyla (i.e. *Ascomycota, Basidiomycota* and *Zygomycota*) because they are widely distributed in soils with high relative abundance^28^. PyOM addition significantly decreased the relative abundance of *Acidobacteria (p* < 0.05) in rhizosphere and bulk soil (Fig. 2A), while it significantly increased the relative abundance of *Bacteroidetes (p* < 0.05) in rhizosphere and bulk soil (Fig. 2B). PyOM also increased the relative abundances of *Actinobateria* and *Alphaproteobacteria* in rhizosphere soil (*p* < 0.05) (Fig. 2C,D). PyOM significantly decreased the relative abundance of *Betaproteobacteria* in bulk soil (*p* < 0.05) and increased it in rhizosphere soil (*p* < 0.05) (Fig. 2E). However, there was no apparent pattern observed in the relative abundances of *Firmicutes* (Fig. 2F). Regarding fungal phyla, PyOM did not significantly change the relative abundances of *Ascomycota, Basidiomycota* and *Zygomycota (p* > 0.05) in either rhizosphere or bulk soils (Fig. 2G–I), except *Ascomycota* and *Basidiomycota* in the treatments of RhMa and RhSt, respectively.

### Community of bacterial and fungal responders

**Figure 3:**
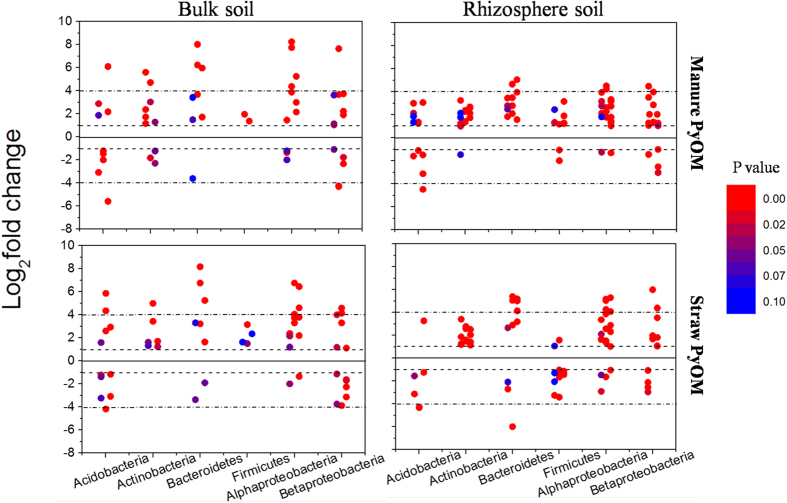
Bacterial responding genera (defined as log2-fold change >1, adjusted p values < 0.1) in each taxonomy as compared with PyOM unamended soils in bulk and rhizosphere soils. Each circle represents a single bacterial genus. Dash line and dash dot line represent increases/decreases of 2x and 16x, respectively. Responders with log2-fold change <1 or adjusted p values > 0.1 were not presented.

**Figure 4:**
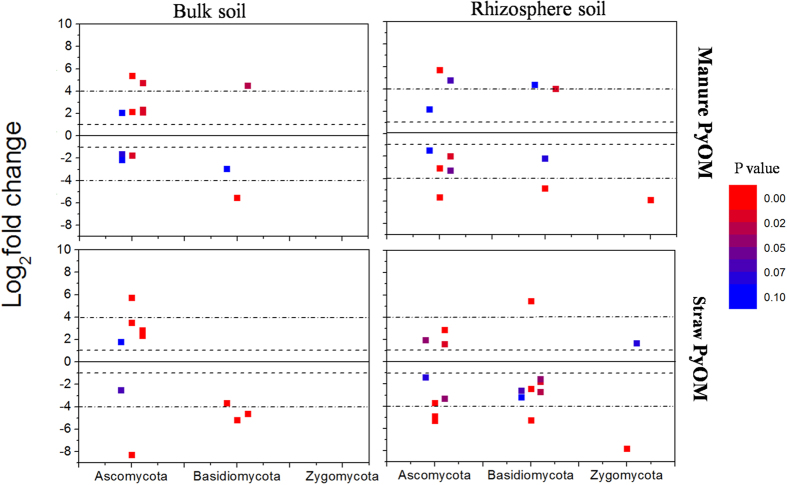
Fungal responding genera (defined as log2-fold change >1, adjusted p values < 0.1) in each phylum as compared with PyOM unamended soils in bulk and rhizosphere soils. Each circle represents a single fungal genus. Dash line and dash dot line represent increases/decreases of 2x and 16x, respectively. Responders with log2-fold change <1 or adjusted p values > 0.1 were not presented.

**Figure 5:**
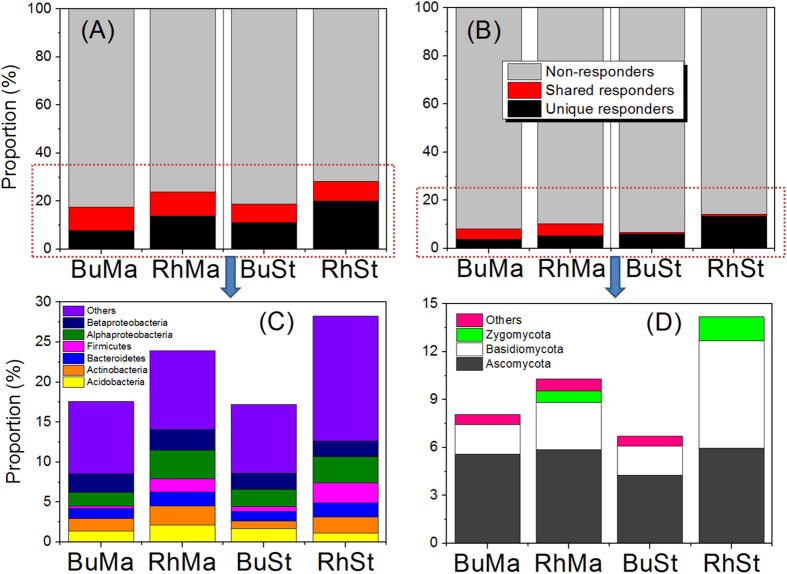
Proportion of responding genera and non-responding genera in bulk and rhizosphere soils with manure and straw PyOM addition. Shared responders represent the responders that were found in both bulk and rhizosphere soils with manure/straw PyOM addition. Unique responders represent the responders that were only found in either bulk or rhizosphere soil with manure/straw PyOM addition.

Regarding that the changes in relative abundance of microbes at coarse taxonomic resolution (i.e. phylum) only provided limited information of how PyOM affected soil microbial community, we identified the microbes whose relative abundance increased/decreased significantly by more than doubling (herein called “responders”) in response to PyOM additions at finer taxonomic resolution (i.e. genus). By this method, we can gain the number and proportion of all the responders that are sensitive to PyOM addition, and identify the classification at phylum level that these responders belong to. Results showed that a large number of responders at genus level, responded significantly to PyOM addition in both rhizosphere and bulk soils (Figs 3 and 4). With bacteria, we identified 113 and 148 responders in the BuMa and RhMa samples, and 112 and 172 responders in the BuSt and RhSt samples, respectively (Fig. 3). The responders whose relative abundance increased/decreased significantly following PyOM addition were 68/45, 101/47, 73/39 and 86/86 in the BuMa, RhMa, BuSt and RhSt samples, respectively (Fig. 3). We also found that the number of *Firmicutes* in rhizosphere soil was notably higher than those in bulk soil, regardless of PyOM types (Fig. 3). With fungi, we identified 13 and 14 responders in the BuMa and RhMa samples, and 11 and 19 responders in the BuSt and RhSt samples, respectively (Fig. 4). The responders whose relative abundance increased/decreased significantly following PyOM addition were 7/6, 6/8, 5/6 and 5/14 in the BuMa, RhMa, BuSt and RhSt samples, respectively (Fig. 4). *Zygomycota* was observed in rhizosphere soil but not in bulk soil (Fig. 4). T-student test showed there was no significant difference in average log_2_fold change of all responders between rhizosphere and bulk soils (*p* > 0.05) (Table S2). Paired t-student test showed that the proportion of responders in bacterial community (ranging from 17.6% to 28.2%) was significantly higher than those in fungal community (ranging from 6.7% to 14.1%) (*p* < 0.05) (Fig. 5). The proportion of responders in rhizosphere soil was also higher than those in bulk soil, regardless of PyOM types and microbial domains (Fig. 5); the bacterial proportion of responders from *Actinobacteria, Bacteroidetes, Firmicutes, Alphaproteobacteria* and other responding taxa in rhizosphere soil was higher than those in bulk soil, and the fungal proportion of responders from *Ascomycota, Basidiomycota and Zygomycota* in rhizosphere soil was also higher than those in bulk soil, regardless of PyOM types (Fig. 5C,D).

### Relationship between environmental parameters and responder community composition

**Figure 6:**
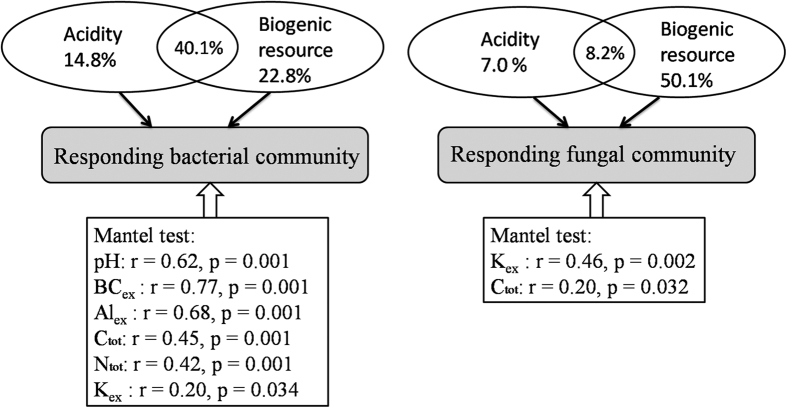
Variation partition analysis (VPA) and Mantel test of the relationships between PyOM-induced parameters (i.e. acidity parameters and biogenic resource parameters) and microbial responder community. The “r” in Mantel test represents the correlation coefficient between environmental and microbial matrices; the “p” value below 0.05 represents the significant difference between environmental parameter and microbial community structure.

For the bacterial responder community composition, variation partition analysis (VPA) showed that the PyOM-induced acidity parameters explained 14.8% of the total variation and the biogenic resource explained 22.8% (Fig. 6). Two parameters co-explained 40.1% of the total variation. Mantel test showed that the important parameters that significantly determined bacterial responder community structure were pH (r = 0.62, *p* = 0.001), BC_ex_ (r = 0.77, *p* = 0.001), Al_ex_ (r = 0.68, *p* = 0.001), C_tot_ (r = 0.45, *p* = 0.001), N_tot_ (r = 0.42, *p* = 0.001) and K_ex_ (r = 0.20, *p* = 0.034)(Fig. 6). The relative abundance of responders in *Actinobacteria* and *Bacteroidetes* was significantly negatively correlated with acidity parameters (e.g. pH, BC_ex_ and Al_ex_) and positively with biogenic resource parameters (e.g. C_tot_, N_tot_) (*p* < 0.05) (Table S3). In contrast, the trend in *Acidobacteria* was opposite (Table S3).

For the fungal responder community, the PyOM-induced biogenic resource parameters explained 50.1% of the total variation and the acidity parameters only explained 7.0%. Two parameters co-explained 8.2% of the total variation. The important parameters that significantly determined fungal responder community structure were K_ex_ (r = 0.46, *p* = 0.002) and C_tot_ (r = 0.20, *p* = 0.032) (Fig. 6). Although the relative abundance of responders in *Ascomycota, Basidiomycota* and *Zygomycota* had no significant correlation with C_tot_ (data not shown), while the number of responders at genus level whose relative abundance significantly correlated with C_tot_ was the highest compared to other biogenic resource parameters (Fig. S3).

## Discussion

### Responder taxa

In general, specific bacterial taxa that are numerically abundant in soil have been classified into two ecological categories of copiotroph and oligotroph^29^. Copiotrophs, e.g. *Bacteroidetes*, preferentially consume soil organic C and have high nutrient requirements, and are more abundant when biogenic resources increase. In contrast, oligotrophs, e.g. *Acidobacteria,* exhibit high abundance in conditions of low nutrient status due to their higher substrate affinities^30^. Our study showed that soil favored more copiotrophs over oligotrophs following PyOM addition: (1) the relative abundance of *Bacteroidetes* increased while *Acidobacteria* decreased significantly (Fig. 2), and (2) the biogenic resource parameter had positive and negative relationships with the responder abundance in *Bacteroidetes* and *Acidobacteria*, respectively (Table S3). This might be due to the direct effect of PyOM, since the PyOMs used in the present study had relatively large fractions of easily mineralizable C (showed by FTIR in Fig. S2) and mineral nutrients^31^ (Tables 1 and S1 and Fig. S1) that can be directly utilized by microbes.

On the other hand, the soil pH was increased following the addition of PyOM (Table 2), and the PyOM-induced pH showed a strong correlation with the relative abundance of *Acidobacteria* (negative) and *Bacteroidetes* (positive)(Table S3). This was coincident with the reports of some previous studies shown that soil acidity parameters, especially soil pH, had the same correlations with *Bacteroidetes* and *Acidobacteria* abundance^32^,^33^. Giving that PyOM can impact soil microbes indirectly though increasing soil biogenic resource availability via modifying soil abiotic factors (e.g. soil acidity correction) or via releasing organic C and mineral nutrients into surrounding soils^34^, this indicated that the indirect effect of PyOM also played an important role in dominant bacterial taxa. Overall, both indirect and direct effects of PyOM were speculated to stimulate copiotrophs growth and inhibit oligotrophs growth in soils in this study. The potential mechanism of PyOM effects on the relative abundance of dominant bacterial taxa was described as follows: PyOM improved the microbial living environment via increasing biogenic resources and correcting soil acidity, and via direct nutrient provision. Additionally, considering that PyOM is porous, the direct effect of PyOM might also be involved by serving as a habitat for microbes to live. However, it was difficult to identify this contribution in the changes of bacterial abundance in the present study. Further investigation could be conducted using PyOMs with a gradient of average pore size.

Here, we should point out the effects of PyOM on the relative abundance of dominant fungal phyla had no apparent trends (Fig. 2). In addition, the relative abundance of fungal responders at phylum level had no significant correlation with PyOM-induced parameters (except K_ex_). However, these results only suggest PyOM had few effects on the overall abundance of fungal groups at coarse taxonomic resolution. At finer taxonomic resolution (i.e. genus), the trend of how PyOM affected soil microbial abundance was more apparent. For instance, at genus level, the relative abundance of 9 responding genera had significant corrections with C_tot_ (Fig. S3), with 5 of them negatively correlated with C_tot_ and 4 of them positively correlated with C_tot_ (Fig. S3). This probably gave a good explanation that why the overall abundance had no significant correlation with PyOM-induced C_tot_ at phylum level.

### Responder community composition

We used the ‘responder community’ to substitute “whole community” for further analysis because there was a large fraction of non-responders that were strongly resistant to PyOM addition. Thus, after the non-responder effect was ruled out, it is more valuable, representative and convincing to investigate the mechanisms why these specific responding groups are so sensitive to PyOM addition. With respect to native soil microorganisms, environmental factors that principally control the microbial community have been widely studied^33^,^35^,^36^. For instance, Lauber *et al*.^35^ consistently reported that how soil pH was the most important parameter in regulating bacterial community structure while nutrient status was most closely associated with fungal communities in arable soils. However, the principal PyOM-induced parameters determining soil microbial community are still poorly understood. As PyOM can strongly change the pH, organic matter and nutrients status of microbial living environment by both direct and indirect effects, the principal parameters determining bacterial and fungal community structures, especially of the sensitive responders, following PyOM addition may be very distinct from natural soils. In this study, it is a great challenge to discern that the chemical parameters measured was derived from indirect effect or direct effect. Therefore, we used the term “PyOM induced-parameters” to cover both direct and indirect effects of PyOM in this section.

For the bacterial responder community, we found that PyOM-induced acidity (54.9%) had the similar contribution to the community structure with biogenic resource (62.9%) (Fig. 6). In addition, the correlation coefficients (r) with pH and C_tot_ were 0.62 and 0.45, respectively, which were different from the previous study demonstrating that pH alone explained the largest fraction of the variability (r = 0.79) in bacterial community determination while the r with C was only 0.23^32^. The mechanisms might be due to the unique properties of PyOM, i.e. the high alkalinity^37^ and abundant labile C^31^. As bacteria are suppressed under acidic conditions^38^, they are probably more susceptible to any environmental disturbance in soil acidic status (e.g. pH, BC_ex_ and Al_ex_). Thus, the addition of PyOM can easily alter the bacterial responder community by decreasing the soil acidity, e.g. pH, BC_ex_ and Al_ex_ (Table 2). Meanwhile, the nutrient status in acidic soil is also very low, and in this situation the bacteria lack substrates to grow, reproduce and metabolize. Following PyOM addition, the quantity and bioavailability of essential substrates (e.g. C_tot_, N_tot_ and K_ex_) were enhanced, which can greatly facilitate the bacterial growth. This hypothesis has been well supported by Liu *et al*.^39^, who demonstrated that the bacterial community was dominated by both soil pH and total C in black soils, of which the average C content (23.9 g kg^−1^) were as high as the PyOM-amended soils in our study (19.6 g kg^−1^).

For the fungal responder community, PyOM-induced biogenic resource itself (50.1% of total variation) was the dominant parameter structuring fungal responder community (Fig. 6), which explained more than 27% of total variation compared to bacterial responder community. This result is supported by Lauber *et al*.^35^, who stated that nutrient content was most closely associated with the fungal community in arable soils. Normally, fungi are more resistant to environmental disturbance than bacteria, and therefore likely readily adapt to environment changes^40^, e.g. acidity change. This speculation was also supported by our results showing that the proportion of responders in fungal community was lower than in bacterial community in the same treatment (Fig. 5). However, fungi are heterotrophs that are sensitive to nutrient status because nutrient status (C and minerals) is the essential substrate for fungal growth and metabolism^41^, especially in an low quality acidic soil. This result is consistent with a previous study revealing that the fungal community was largely driven by soil carbon content^42^ and with our results showing that fungal responder community structure were significant correlated with K_ex_ (r = 0.46, *p* = 0.002) and C_tot_ (r = 0.20, *p* = 0.032)(Fig. 6). In conclusion, multivariate analyses revealed that PyOM-induced acidity and biogenic resource co-determined the bacterial community structures, while biogenic resource was the dominant parameter structuring fungal responder community. The differences were likely attributed to the different physiological traits and ecological lifestyles.

### Differences between rhizosphere and bulk soils

In this study, the number and proportion of responders in rhizosphere soil were larger than in bulk soil, regardless of PyOM types and microbial domains (Figs 3, 4 and 5). The similar trend was also observed in the proportion of dominant bacterial taxa and fungal phyla (Fig. 5), indicating both bacterial and fungal community in rhizosphere soil was more sensitive to PyOM addition than in bulk soil. Here, we found that PyOM addition significantly increased the plant root length and root biomass (Fig. 1), consistent with the previous findings suggesting that PyOM addition stimulated root growth^43^,^44^. This might be attributed to both the direct and indirect effects of PyOM. The potential direct mechanisms were: (1) the nutrients (organic C and minerals) in PyOM (Tables 1 and S1, Figs S1 and S2) can act as the “fertilizer” and be directly utilized by plant root^8^; (2) the specific pore structures of PyOM (Fig. S1) can induce root hair formation^45^. The indirect mechanisms may include, but are not limited to: (1) reduced soil density and tensile strength caused by PyOM which can relieve physical constraints^46^; (2) increased water holding capacity induced by PyOM^47^, which alleviates drought stress for root growth; (3) the reduced soil reflectivity and warmed soil temperature caused by PyOM^48^, which can strongly affect root growth^49^; (4) corrected soil abiotic factors by PyOM (e.g. pH change), which can indirectly increase nutrient availability^50^. Worth mentioning was that, the above mechanisms for driving changes in root growth were most likely to act concurrently; due to the complexity of PyOM-root-soil system, it was a great challenge to distinguish any indirect effect from the direct one. However, based on the present observation, together with the previous findings, there seems to be a common perception that PyOM addition increased root growth and development.

Generally, root growth and elongation are accompanied by rhizo-deposition, i.e. the release of carbon exudates into rhizosphere soil^51^,^52^. The carbon exudates would probably stimulate microbes to proliferate and metabolize, resulting in higher number and proportion of responders in rhizosphere soil (Fig. 5), as both bacterial and fungal communities were strongly determined by biogenic resource parameter. Moreover, the BET in straw and manure PyOMs were 52.6 m^2^ g^−1^ and 11.7 m^2^ g^−1^, respectively (Table 1), and there were some pore structures in PyOMs (Fig. S1). Due to its adsorption capacity and high pore structure^53^, PyOM may probably retain the easily degradable C exudates and protect them from leaching or diffusion into surrounding soils^54^, making C substrates in rhizosphere more accessible for microbes. This was supported by the phenomenon that the straw PyOM with higher surface area and more pore structures resulted in higher proportion of responders in rhizosphere soils (RhSt)(Fig. 5), compared to manure PyOM (RhMa). Thus, the PyOM mediated root processes, namely, PyOM modified rhizosphere effect, were PyOM-feedstock dependent, and were likely the important mechanism explaining the different patterns between rhizosphere and bulk soils. However, it should be pointed out that in most cases the difference of PyOM effects on root growth, microbial taxa and microbial responders were not significant between manure and straw PyOM. This indicated that the effects of PyOM on microbial abundance and diversity were not strongly determined by feedstock types of PyOM. Based on our results, the mineral nutrients in manure PyOM were higher than in straw PyOM, while the organic C and pore structures had the opposite trends. This would lead to mutually offsetting effect of PyOM feedstock types on root growth and microbial community structures. To be specific, with respect to the facilitated root growth and changes in microbial community, the contribution of mineral nutrients in manure PyOM was likely higher than that in straw PyOM, while the contribution of organic C and pore structures in straw PyOM might be higher than that in manure PyOM.

On the other hand, although the number of responders in rhizosphere soil was higher than in bulk soil, t-student test showed that the average log_2_fold change of responders between rhizosphere and bulk soils was not significant (Table S2). This suggested PyOM addition could only induce more sensitive microbes in rhizosphere soil, while the abundance change of these responders in rhizosphere soil was similar to bulk soil. We also found that the specific responders in rhizosphere soil were *Firmicutes* for bacteria and *Zygomycota* for fungi, which can be used as the indicator species in the future study. Future investigations should be conducted targeting of their ecological roles associated with PyOM addition to rhizosphere soil.

In summary, selection of responding taxa at genus level from the whole microbial community made it possible to get more in-depth information of how PyOM affects soil microbial community. PyOM changed the soil microbial abundance and community structure through altering microbial living environment, via their indirect effects by improving soil chemical parameters (e.g. acidity and biogenic resource), and via direct effects due to its unique properties (e.g. sufficient nutrients and pore structures). The significant increased root growth caused by PyOM addition, associated with physiological processes, e.g. rhizodeposition, likely favored more sensitive responders in rhizosphere than in bulk soil.

## Materials and Methods

### PyOM preparation and soil collection

We collected the feedstocks of swine manure and rice straw from a hoggery and a farmland in Hangzhou, China, respectively. To produce PyOMs, feedstocks were pyrolyzed in a modified furnace under anaerobic conditions at the pyrolysis temperature of 550 °C for 2 hours. The basic properties of manure and straw PyOMs are presented in Tables 1 and S1, Figs S1 and S2. We also collected the paddy soil (classified as Inceptisol in USDA Soil Taxonomy) with a pH of 5.35 and total organic carbon of 10.7 g kg^−1^ from a low yield, acidic, paddy soil field (0–20 cm depth) in Zhejiang, China (119.47°N, 29.02°E). It was then air-dried, crushed and sieved <2 mm for pot experiment. The methods for PyOM characterization was described in the Supporting Information.

### Pot experiment

Three treatments (manure PyOM added at 3%, straw PyOM added at 3%, and the control soil without PyOM addition) were prepared in triplicate, and each pot, 28 × 24 cm, was loaded with 4 kg soil. We added fertilizers to each pot and also intimately mixed them with PyOM-soil mixtures. The fertilizers added were: urea, 650 mg kg^−1^; potassium dihydrogen phosphate, 438 mg kg^−1^; potassium chloride, 335 mg kg^−1^. The rice seedlings were planted and cultivated in each pot in a greenhouse. After maturity, rhizosphere (Rh) and bulk (Bu) soils were sampled from each treatment separately according to the method of Riley and Barber^55^. The abbreviations for soil samples are described as follows: BuCK (no PyOM in bulk soil), BuMa (manure PyOM in bulk soil), BuSt (straw PyOM in bulk soil), RhCK (no PyOM in rhizosphere soil), RhMa (manure PyOM in rhizosphere soil) and RhSt (straw PyOM in rhizosphere soil). Samples used for chemical analyses were stored at −20 °C, and others were stored at −80 °C for DNA extraction, amplification and high throughput sequencing.

### Soil chemical analysis

Soil total C (C_tot_) and total N (N_tot_) were determined using a Flash EA 1112 elemental analyzer (Thermo Scientific, USA). Soil dissolved organic carbon (DOC) was determined using a TOC/TN analyzer (Analytik Jena AG, Germany). Soil pH was determined using a pH meter at a 1:2.5 (w/w) soil:water ratio. Exchangeable Ca^2+^, Mg^2+^, K^+^ and Na^+^ were extracted with 1 M ammonium acetate at pH 7.0. The total exchangeable base cations (BC_ex_) were calculated from the sum of exchangeable Ca^2+^, Mg^2+^, K^+^ and Na^+^, which were determined by flame atomic absorption spectrometry (Analytikjena, Germany). The concentration of soil exchangeable Al (Al_ex_) was calculated from the difference between the exchangeable acidity and hydrogen, which were measured by NaOH auto titration. Soil available P (P_avail_) was extracted with 0.5 mol L^−1^ NaHCO_3_ at pH 8.5 and then measured using the Mo-Sb colorimetric method. The root length was carefully measured by hand with a ruler and the root biomass was determined as described by Noguera *et al*.^56^.

The pH, BC_ex_ and Al_ex_, which are related to soil acidity, were further grouped as PyOM-induced acidity parameters, while the C_tot_, N_tot_, P_avail_, K_ex_ and DOC, which are essential to microbial growth and metabolism, were further grouped as PyOM-induced biogenic resource parameters.

### DNA extraction and Illumina Sequencing

Samples collected from rhizosphere and bulk soils were both used for DNA extraction, and then for Illumina Miseq sequencing. The bacterial and fungal DNA were extracted from 0.25 g soil samples using a MoBioPowerSoil^®^ DNA isolation kit (MoBioLaborarories, USA), following the manufacturer’s instructions. The V4 region of the 16S rRNA gene in each bacterial sample was amplified by the polymerase chain reaction (PCR). The sequences of the forward and reverse primers were 515F: 5′-GTGCCAGCMGCCGCGGTAA-3′ and 806R: 5′-GGACTACHVGGGTWTCTAAT-3′, respectively (provided by the Beijing Genomics Institute, China). For fungal genes, the ITS1 region was amplified by the PCR. The forward and reverse primers were its1: 5′-CTTGGTCATTTAGAGGAAGTAA-3′ and its2: 5′-GCTGCGTTCTTCATCGATGC-3′, respectively (provided by the Beijing Genomics Institute, China). The procedures for bacterial and fungal DNA amplification were conducted by the Beijing Genomics Institute, China. Each sample was amplified in triplicate, and the PCR products were then purified using MinElute PCR purification Kit (Qiagen, Germany), and sequenced with a MiSeq sequencing platform (Illumina, USA).

### Processing sequencing data

Low-quality reads were firstly removed from the raw data with two paired-end reads^57^. Then, the paired end reads were merged to tags by FLASH^58^, and the detailed method is described as follows: (1) Minimal overlapping length: 15 bp; (2) Mismatching ratio of overlapped region: < = 0.1. Operational taxonomic units (OTUs) at a 97% similarity level were clustered using the Usearch (version 7.1 http://drive5.com/uparse/)^59^ and the taxonomy was assigned based on Greengenedatabase^60^ for bacteria and UNITE database^61^ for fungi. Also, the chimera check was performed during the process^62^. All sequences have been deposited in the GenBank short-read archive SRP073769.

### Community analysis

We tested the significant differences (*p* < 0.05) in (1) chemical parameters of PyOM amended soils; and (2) relative abundances of the dominant bacterial and fungal taxa, by general analysis of variance (ANOVA). R package ‘DESeq2’^63^ was used to calculate the differential abundance (log2-foldchange in relative abundance of each genus) for each PyOM amended sample as compared with control^26^. The P-values was adjusted by the BH (Benjamini andHochberg) correction method and a FDR (false discovery rate) of 10% was used to denote statistical significance. Finally, we selected the responders with log2-fold change in relative abundance >1 and an adjusted P-value of <0.1 for further community analysis. We conducted t-student test to analyze the significant differences (p < 0.05) in average log_2_fold change of whole responder community between rhizosphere and bulk soils. To investigate the relationships between PyOM-induced chemical parameters and PyOM-induced microbial responder community structure, variation partitioning analysis (VPA) was conducted using the “vegan” package in R software^64^. The Mantel test was performed to examine the principal PyOM-induced chemical parameter in determining the responder community structure. Pearson’s rank correlation was performed to match the relative abundance of dominant responders with PyOM-induced chemical parameters.

## Additional Information

**How to cite this article**: Dai, Z. *et al*. Sensitive responders among bacterial and fungal microbiome to pyrogenic organic matter (biochar) addition differed greatly between rhizosphere and bulk soils. *Sci. Rep.* **6**, 36101; doi: 10.1038/srep36101 (2016).

**Publisher’s note**: Springer Nature remains neutral with regard to jurisdictional claims in published maps and institutional affiliations.

## Supplementary Material

Supplementary Information

## Figures and Tables

**Table 1:** Basic properties of manure PyOM and straw PyOM^a^.

**PyOM**	**pH**	**Ash (%)**	**Total C (%)**	**Total H (%)**	**Total N (%)**	**BET (m**^**2**^ **g**^**−1**^)	**Total P (mg kg**^**−1**^)	**BC**_**ex**_ **(cmol kg**^**−1**^)
Manure	9.55	68.3	21.5	1.11	1.3	11.7	33297	8.2
Straw	10.09	39.2	47.0	1.87	1.4	52.6	3068	10.6

^a^BET, specific surface area; BC_ex_, exchangeable base cations.

**Table 2:** Acidity parameters (pH, BC_ex_ and Al_ex_) and biogenic resource parameters (C_tot_, N_tot_, P_avail_, K_ex_, DOC) in soils following addition of manure and straw PyOMs at 3%.

	**pH**	**BC**_**ex**a_**(cmol kg^−1^)**	**Al**_**ex**_**(cmol kg^−1^)**	**C**_**tot**_ **(g kg^−1^)**	**N**_**tot**_ **(g kg^−1^)**	**P**_**avail**_ **(mg kg^−1^)**	**K**_**ex**_ **(cmol kg^−1^)**	**DOC (mg kg^−1^)**
BuCK	5.33 a^b^	0.39 a	0.74 b	11.81 a	0.99 a	11.56 a	0.03 a	52.3 a
BuMa	6.64 c	1.18 b	0.00 a	19.82 b	1.40 b	37.34 b	0.08 a	95.0 b
BuSt	5.99 b	0.96 b	0.08 a	28.54 c	1.29 b	13.08 a	0.24 b	55.7 a
RhCK	5.13 A	0.29 A	0.93 B	12.58 A	0.93 A	12.91 A	0.03 A	67.6 A
RhMa	6.94 C	1.34 C	0.07 A	18.56 B	1.40 B	32.89 B	0.08 B	88.1 B
RhSt	6.11 B	1.05 B	0.02 A	26.43 C	1.23 AB	11.62 A	0.34 C	83.1 B

^a^BC_ex_, total exchangeable base cations; Al_ex_, exchangeable Al; C_tot_, total C; N_tot_, total N; P_avail_, available phosphorus; K_ex_, exchangeable K; DOC, dissolved organic carbon.

^b^Different letter in lower case within the same column indicates that the difference is significant at p < 0.05 probability level in bulk soil samples; Different letter in upper case indicates that the difference is significant at p < 0.05 probability level in rhizosphere soil samples.
