# Supplementary material for: Sensitive responders among bacterial and fungal microbiome to pyrogenic organic matter (biochar) addition differed greatly between rhizosphere and bulk soils
Source: Sci Rep. 2016 Nov 8;6:36101. doi: 10.1038/srep36101 (PMC5099700; doi:10.1038/srep36101)

**Sensitive responders among bacterial and fungal microbiome to pyrogenic organic matter addition differed greatly between rhizosphere and bulk soils**

Zhongmin Dai^1,2^, Jiajie Hu^1,2^, Xingkun Xu^1,2^, Lujun Zhang^1,2^, Philip C. Brookes^1,2^, Yan He^1,2^*, Jianming Xu^1,2^

^1^Institute of Soil and Water Resources and Environmental Science, College of Environmental and Resource Sciences, Zhejiang University, Hangzhou 310058, China

^2^Zhejiang Provincial Key Laboratory of Agricultural Resources and Environment, Hangzhou 310058, China

* Corresponding author. Y. He, College of Environmental and Resource Sciences, Zhejiang University, 866 Yuhangtang Road, Hangzhou 310058, China. Email: yhe2006@zju.edu.cn; Tel/fax: +86-571-88982065.

**PyOM characterization.**

Ash content was determined as follows: 1.00 g of the ground biochar was heated at 700 °C for 2 h in a muffle furnace, and the ash (%) was calculated from ash (%) = (weight of ash)/(weight of biochar) × 100. pH of PyOMs was determined in deionized water at the ratio of 1:10 w/w biochar/water. Total C, H, and N concentrations of PyOMs were measured with a Flash EA 1112 elemental analyzer (Thermo Scientific, USA). The P, base cations (K, Na, Ca, and Mg), Fe, Cu and Zn were analyzed following digestion PyOMs with H_2_O_2_ and HNO_3_ (1:5) at 120 °C for 4 h, then adjusted to 50 ml with deionized water. The total P was then determined using a continuous colorimetric flow system (SkalarSAN++ System, the Netherlands). The total base cations (Ca, Mg, K and Na), Fe, Cu and Zn concentrations were determined by a NovAA300 atomic absorption spectrometer (Analytikjena, Germany). Exchangeable base cations (K, Na, Ca, and Mg) concentrations were extracted by 1.0 mol/L ammonium acetate using a ratio of 1:10 w/v PyOM/ solution and then measured by atomic absorption spectrometer (Analytikjena, Germany). Surface area (BET) was measured using a Nova 2200e surface area analyzer (Quantachrome, USA) after degassing at 200 °C for 8 h. Morphology and nutrient content of PyOMs (coated with Pt) were determined by a Sirion 100 thermal field emission scanning electron microscope (FEI, Netherlands) equipped with a Genesis 4000 X-ray energy dispersive spectroscope (EDAX, USA) (SEM-EDS). Functional groups on PyOM surface was measured by An Avatar370 Fourier transform infrared (FTIR) spectrometer with a Nicolet 380 spectrophotometer (ThermoNicolet, USA).

Table S1. Total concentrations of K, Na, Ca, Mg, Fe, Cu and Zn of manure PyOM and straw PyOM.

| PyOM | K  (mg kg^-1^) | Na  (mg kg^-1^) | Ca  (mg kg^-1^) | Mg  (mg kg^-1^) | Fe  (mg kg^-1^) | Cu  (mg kg^-1^) | Zn  (mg kg^-1^) | Total base cations  (mg kg^-1^) |
| --- | --- | --- | --- | --- | --- | --- | --- | --- |
| Manure | 30256 | 10153 | 23718 | 19047 | 12677 | 2382 | 2428 | 83174 |
| Straw | 45749 | 9404 | 11098 | 4234 | 1015 | 46 | 257 | 70485 |

Table S2. T-student test of the average relative abundance of the whole responder community between rhizosphere and bulk soils.

| Microbial type | PyOM type | Soil type | Log_2_fold change | p |
| --- | --- | --- | --- | --- |
| Bacteria | Manure | Rhizosphere | 3.13 | p>0.05 |
|  |  | Bulk | 3.00 |  |
|  | Straw | Rhizosphere | 2.35 | p>0.05 |
|  |  | Bulk | 2.34 |  |
| Fungi | Manure | Rhizosphere | 3.18 | p>0.05 |
|  |  | Bulk | 4.14 |  |
|  | Straw | Rhizosphere | 3.95 | p>0.05 |
|  |  | Bulk | 3.29 |  |

Table S3. Correlations between relative abundances of bacterial responders and PyOM-induced soil parameters. **: *p*<0.01; *: *p*<0.05.

|  | pH | BC_ex_ | Al_ex_ | P_avail_ | K_ex_ | C_tot_ | N_tot_ | DOC |
| --- | --- | --- | --- | --- | --- | --- | --- | --- |
| *Actinobacteria* | 0.81^**^ | 0.90^**^ | -0.78^**^ | 0.56^*^ | - | 0.56^*^ | 0.68^**^ | 0.48^*^ |
| *Acidobacteria* | -0.89^**^ | -0.91^**^ | 0.94^**^ | -0.49^*^ | -0.56^*^ | -0.72^**^ | -0.66^**^ | -0.494^*^ |
| *Bacteroidetes* | 0.61^**^ | 0.76^**^ | -0.79^**^ | - | 0.81^**^ | 0.76^**^ | 0.55^*^ | - |
| *Alphaproteobacteria* | - | - | - | - | - | - | 0.56^*^ | - |
| *Firmicutes* | - | - | - | 0.63^**^ | - | - | - | 0.70^**^ |
| *Betaproteobacteria* | - | - | - | - | - | - | - | - |

Fig. S1. SEM-EDS analysis of manure PyOM and straw PyOM. (A) SEM spectrum of manure PyOM; (B) SEM spectrum of manure PyOM; (C) EDS spectrum of manure PyOM; (D) EDS spectrum of manure PyOM;


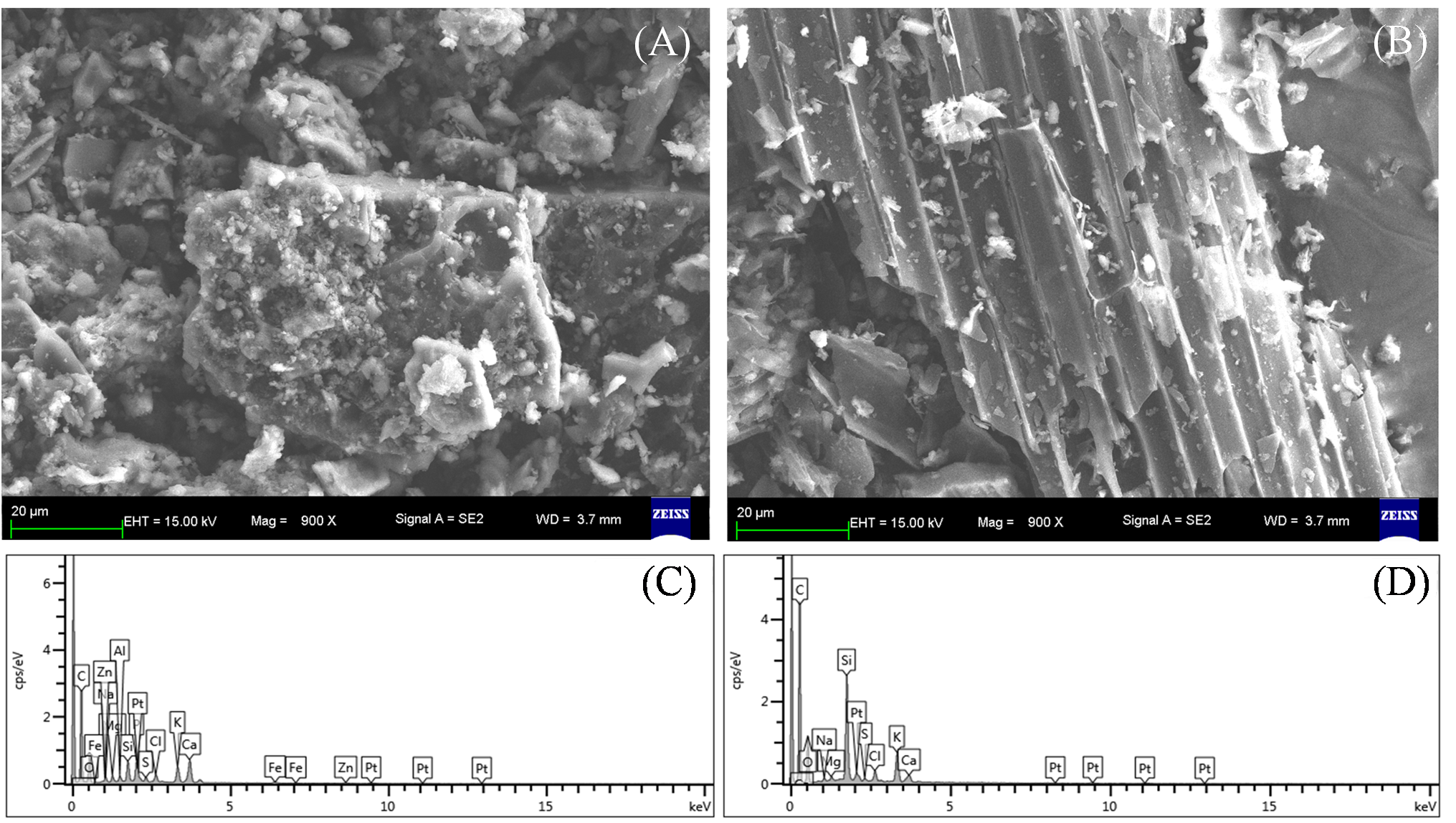


Fig. S2. FTIR spectra of manure PyOM and straw PyOM.


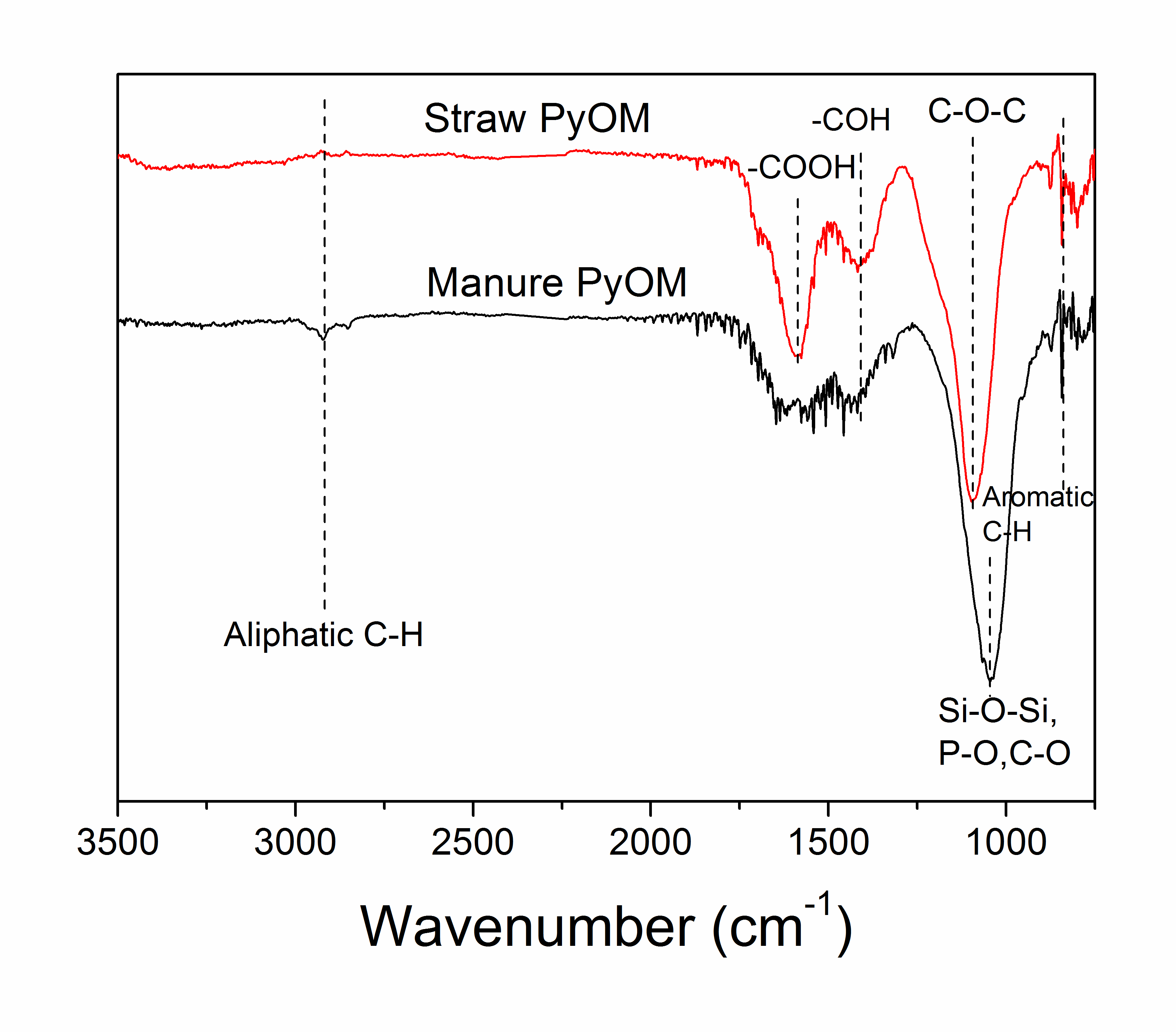


Fig. S3. Number of fungal responders at genus level whose relative abundance significantly correlated with biogenic resource parameters (C_tot_, N_tot_, K_ex_, P_avail_ and DOC).


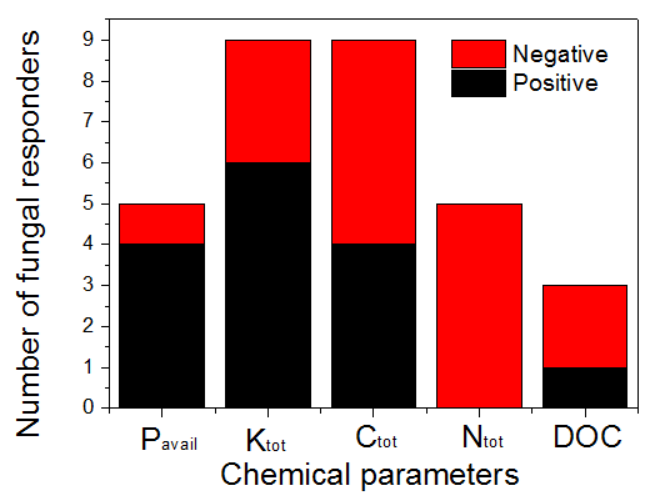

Supplement: Supplementary Information [file srep36101-s1.doc]
